# Contact tracing reveals community transmission of COVID-19 in New York City

**DOI:** 10.21203/rs.3.rs-1840065/v1

**Published:** 2022-07-27

**Authors:** Sen Pei, Sasikiran Kandula, Jaime Cascante Vega, Wan Yang, Steffen Foerster, Corinne Thompson, Jennifer Baumgartner, Shama Ahuja, Kathleen Blaney, Jay Varma, Theodore Long, Jeffrey Shaman

**Affiliations:** Columbia University; Mailman School of Public Health, Columbia University; Columbia University; Columbia University; New York City Department of Health and Mental Hygiene; New York City Department of Health and Mental Hygiene; New York City Department of Health and Mental Hygiene; New York City Department of Health and Mental Hygiene; New York City Department of Health and Mental Hygiene; Weill Cornell Medical College; NYC Health + Hospitals; Mailman School of Public Health, Columbia University

## Abstract

Understanding SARS-CoV-2 transmission within and among communities is critical for tailoring public health policies to local context. However, analysis of community transmission is challenging due to a lack of high-resolution surveillance and testing data. Here, using contact tracing records for 644,029 cases and their contacts in New York City during the second pandemic wave, we provide a detailed characterization of the operational performance of contact tracing and reconstruct exposure and transmission networks at individual and ZIP code scales. We find considerable heterogeneity in reported close contacts and secondary infections and evidence of extensive transmission across ZIP code areas. Our analysis reveals the spatial pattern of SARS-CoV-2 spread and communities that are tightly interconnected by exposure and transmission. We find that higher vaccination coverage and reduced numbers of visitors to points-of-interest are associated with fewer within- and cross-ZIP code transmission events, highlighting potential measures for curtailing SARS-CoV-2 spread in urban settings.

## Introduction

Within metropolitan areas, infection risk and disease burden due to SARS-CoV-2, the causative agent of COVID-19, are characterized by spatial heterogeneity at neighborhood scales^1–3^. Communities with substantial local infections can sustain the spread of SARS-CoV-2, seed infections in interconnected neighborhoods, and spark resurgences of cases following the relaxation of non-pharmaceutical interventions (NPIs), such as masking and social distancing^4^. In densely populated urban settings, public health tactics may need to be uniquely tailored to specific geographic areas and/or communities that most support the persistence and spatial dispersion of SARS-CoV-2 infections. Development of such tailored tactics requires improved understanding of both transmission patterns at fine geographical scales and the factors shaping the intensity of community outbreaks. Examples of previously utilized targeted intervention include limiting indoor dining and gathering, increasing testing availability, encouraging home quarantine for exposed contacts, requiring face masks indoors, and closing nonessential businesses in high-risk communities. While the transmission patterns of SARS-CoV-2 at global, national, and regional levels have been reported^5–12^, research on community-level transmission is often challenging due to limited availability of high-resolution surveillance and testing data, the lack of routine case interviews, and the difficulty identifying transmission events. In addition, the effect of public health interventions on community transmission of SARS-CoV-2 in metropolitan areas has not been well evaluated.

Data collected through contact tracing efforts have provided valuable insights into the transmission dynamics of SARS-CoV-2^13–17^; however, most contact tracing during the early phase of the pandemic mainly focused on specific local outbreaks, which cannot support population-level analysis of community transmission. Here, we use detailed data from confirmed and probable cases^18^ and case investigations during the second pandemic wave in New York City (NYC) to quantify community spread of COVID-19 at small spatial scales from October 2020 to May. Unlike the initial outbreak during the spring of 2020, the second pandemic wave was fully captured by contact tracing. Additionally, contact tracing operation and individual protective measures such as mask-wearing and social distancing remained relatively stable during this period of the pandemic (in contrast with the post-Omicron era when protective measures were largely abandoned). As a result, data collected during the second pandemic wave may better inform understanding of SARS-CoV-2 community transmission in NYC and the operational performance of contact tracing during a public health emergency.

## Results

### Contact tracing in NYC

**Figure 1:**
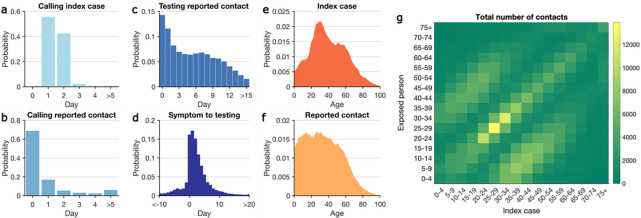
Key statistics of contact tracing in NYC. Panels (a-d) show the distributions of: (a) time between reporting date for index cases and being called by contact tracers; (b) time between calling index cases and notifying exposed persons; (c) time between notifying exposed persons and specimen sampling of notified individuals who were tested; (d) time from symptom onset to specimen sampling for symptomatic COVID infections. A negative value implies that testing preceded symptom onset. Age distributions of index cases (e) and self-reported contacts (f). The contact mixing matrix (g) shows the total number of exposures among age groups reported during the study period.

The NYC Test & Trace Corps initiative was launched in June 2020^19^. Established as an operation to provide contact tracing, testing, and resources to support isolation and quarantine, the contact tracing program was integrated with a set of intervention efforts designed to limit morbidity and mortality from COVID-19 in NYC (Supplementary Information). We analyzed data obtained from case investigations and COVID-19 testing results (molecular and antigen) collected between October 1, 2020 and May 10, 2021 (Extended Data Fig. 1, Supplementary Information). During this period, 691,834 confirmed and probable cases were reported to the New York City Department of Health and Mental Hygiene (DOHMH)^20^. After excluding cases residing in residential congregate settings, cases were sent to the NYC Test & Trace Corps for contact tracing. Among these cases, 644,029 were reached by tracers and 450,415 completed an interview. In total, 779,011 contacts with confirmed and probable cases were self-reported via case investigations, of whom 20.9% (162,659/779,011) were subsequently tested. The median time from specimen collection to reporting results to DOHMH was 2 days. 97% of index patients were called by tracers within two days of reporting to DOHMH (Fig. 1a) and 68.4% of contacts were called the day of reporting to the Test & Trace team (Fig. 1b). Among tested contacts, 66.6% sought testing within one week of notification (Fig. 1d). For traced symptomatic infections, 86.7% were tested after symptom onset, and 13.3% were tested before symptom development (Fig. 1d).

Adults aged 20 to 49 years old constituted the majority of index cases (Fig. 1e), a finding in agreement with the age distribution of confirmed infections in the United States^21^. Self-reported contacts were more uniformly distributed among the population under 50 years old (Fig. 1f). The age-stratified contact matrix highlights more frequent interactions among individuals of similar age and inter-generation mixing within the household (Fig. 1g), a pattern also observed in other countries^22^.

### Exposure and transmission networks

**Figure 2:**
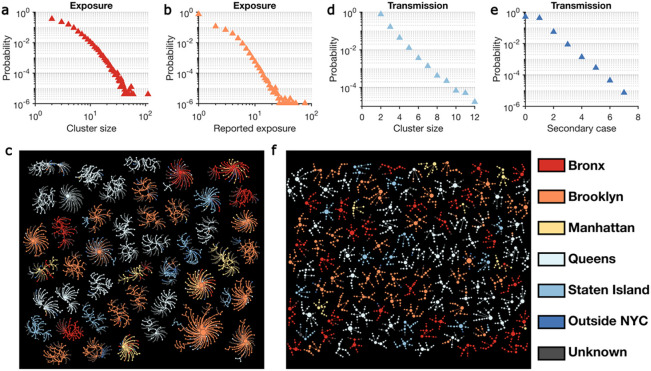
Structure of exposure and transmission networks. (a) and (b) show the distributions of cluster size and number of close contacts reported by each index case in the exposure network. Exposure clusters with more than 35 individuals are visualized in (c). The exposure network is undirected. Index cases and reported close contacts are connected. Node size is proportional to the number of connected individuals. Colors indicate the home location of each person (five boroughs in NYC, outside NYC, and unknown). The distributions of cluster size and the number of secondary cases in the transmission network are shown in (d) and (e), respectively. Panel (f) visualizes transmission clusters with more than six infected individuals. Node size represents the number of secondary cases. Arrows indicate the direction of transmission.

We reconstructed the self-reported exposure network at the individual level for the study period. The exposure network was highly fragmented, with 947,042 individuals in 242,486 disjoint clusters. Cluster size showed considerable heterogeneity (Fig. 2a), as did the number of contacts reported by each index case (Fig. 2b). We visualize several large exposure clusters in Fig. 2c, color-coded by the home borough of each person. Exposure clusters exhibit diverse structures ranging from hub-and-spoke networks with a single spreader to networks with multiple spreaders. Over half of the clusters shown in Fig. 2c were in Queens and Brooklyn. Within the large exposure clusters in Fig. 2c, 1,195 index patients (59.4%) reported contacts living in the same borough, but 817 (40.6%) cross-borough contacts were also recorded.

We further reconstructed transmission chains by linking the contact tracing records and the laboratory-confirmed cases (molecular and antigen). Due to asymptomatic and pre-symptomatic shedding^23–25^, index cases were not necessarily the source of infections in these putative transmission events. To infer the direction of transmission, we estimated the infection date of lab-positive cases. For symptomatic cases, infection date was estimated using an empirical incubation period distribution obtained from a prior study^17^; for asymptomatic cases, we used specimen collection date to estimate infection date using a model of viral load dynamics coupled with a Bayesian inference (Extended Data Fig. 2)^26^. We sampled an ensemble of possible transmission networks compatible with the estimated chronological order of infections and selected the network with maximum likelihood based on transmission probabilities across age groups (Extended Data Table 1, Extended Data Fig. 3). More details on the transmission network reconstruction are provided in the Supplementary Information.

During the study period, we identified 58,474 potential transmission clusters formed by exposures that resulted in lab-confirmed infections. On average, these transmission clusters had a mean size of 2.3 individuals, representing 19.6% (135,478/691,834) recorded cases during the study period. However, transmission cluster size and the number of secondary cases linked to each index case had large variance (Fig. 2d–e) – only 0.20% of transmission clusters involved more than 6 infections. The largest transmission cluster identified consisted of 12 cases, and the maximum number of secondary cases for each single index case was 7. Transmission clusters with at least 6 infections are visualized in Fig. 2f.

**Figure 3:**
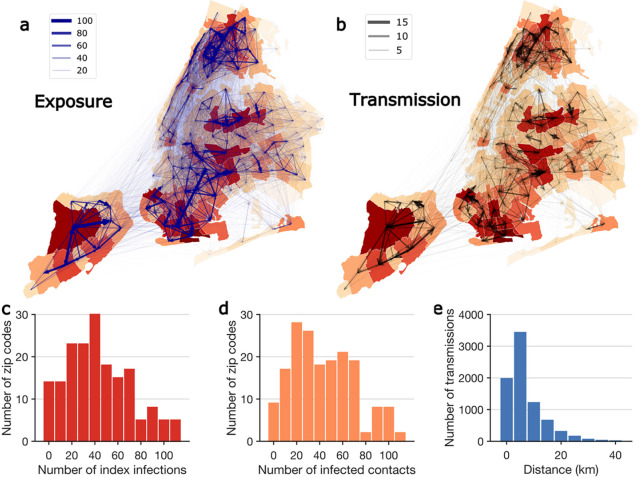
Spatial transmission of SARS-CoV-2 in NYC. (a) and (b) show the exposures and transmission events across ZIP codes in NYC identified from contact tracing data. Arrows indicate direction of exposure (from index cases to reported close contacts) and transmission (from index infections to infected contacts). Arrow thickness indicates the number of exposures and transmission events. ZIP code area color represents the cumulative number of confirmed cases during the study period (yellow to red – low to high). For cross-ZIP code transmission events, the distributions of index infections and infected contacts across ZIP code areas are presented in (c) and (d). Panel (e) shows the distribution of distance between home ZIP codes of index infections and infected contacts in cross-ZIP code transmission events. The population weighted centroids for ZIP code areas were used to compute the distance.

To quantify the spatial spread of SARS-CoV-2 in NYC at fine geographical scales, we mapped exposure and transmission networks across modified ZIP code tabulation areas (MODZCTAs, referred to as ZIP codes hereafter; Fig. 3a–b). Among 72,191 transmission events where place of residence was known, 7,826 (10.8%) included multiple ZIP codes. We observed several local clusters of ZIP codes that were tightly interconnected by exposure and transmission, centered around locations with high community prevalence. Infections in those high-prevalence ZIP code clusters were linked to self-reported contacts in nearby and far locations (Fig. 3a), which may have facilitated the spread of COVID-19 across the city (Fig. 3b). Among the cross-ZIP code transmission chains, we examined distributions of index cases who initiated transmission (Fig. 3c) and the infected contacts (Fig. 3d) across ZIP codes. A distinct skew in the distribution suggests that certain ZIP codes were more involved in the spatial spread of COVID-19. Geographically, most cross-ZIP code transmission events occurred within 10 km; however, long-distance transmission up to 40 km was also evident (Fig. 3e).

### Evaluation of intervention measures

During the period from October 2020 to March 2021, a dynamic zone-based control strategy was adopted in New York State to limit viral spread in communities with high case growth rates while avoiding undue harm to the economy^27^. Three tiers of zones (yellow, orange, and red) were identified based on a set of metrics, collectively defined by test positivity rate, hospital admissions per capita, and hospital capacity^27,28^. Local restrictions on business and services were imposed based on zone conditions. Compliance to these restrictions can be reflected by population mobility in each ZIP code. In December 2020, vaccines became available to the population at highest risk for severe outcomes associated with COVID-19 in NYC and were subsequently available to all eligible individuals over 15 years old during early April 2021. With the support of the detailed contact tracing data, we evaluated the impact of these public health interventions on community transmission of SARS-CoV-2 in NYC.

**Figure 4:**
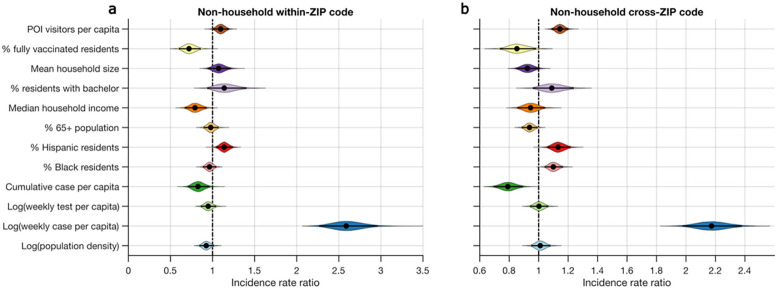
Effects of various features on the transmission of SARS-CoV-2 in NYC. Incidence rate ratios (exponentiated coefficients) for non-household within-ZIP code transmission and cross-ZIP code transmission are shown for 12 covariates in (a) and (b), respectively (Deviance information criterion, DIC=6,342 for a and DIC=12,644 for b). Coefficients were estimated using a Poisson generalized linear mixed model controlling for spatial-temporal autocorrelations. We used the log-transformed population as the offset in the regression model. Covariates were standardized and are shown on the y-axis. The incidence rate ratio quantifies the multiplicative change in the number of transmission events per each covariate increase of one standard deviation, controlling for other covariates. The violin plots show the distributions of incidence rate ratios. Black dots and horizontal black lines highlight the median estimates and 95% CIs.

We assessed the associations of the numbers of non-household within- and cross-ZIP code transmission events across NYC with demographic, socioeconomic, disease surveillance, vaccination coverage, and human mobility features (Supplementary Information). As non-household transmission contributed to the expansion of SARS-CoV-2 outside the household, we focused on 4,642 non-household transmission events. We used aggregated foot traffic records derived from mobile phone data^29^ documenting weekly numbers of individuals visiting points-of-interest (POIs, e.g., restaurants, grocery stores, gyms, and bars) in each ZIP code as an indicator of human mobility and compliance with the zone-based local restrictions (Supplementary Information). We used conditional autoregressive (CAR) models^30^ to assess the effects of the above factors on within- and cross-ZIP code transmission (Fig. 4). Specifically, for both within- and cross-ZIP code transmission, we fitted Poisson generalized linear mixed models (GLMM) with random effects and CAR priors to account for the inherent spatial-temporal autocorrelation in disease transmission data^30,31^ (Supplementary Information, Extended Data Figs. 4–5).

We found that higher vaccination coverage and fewer POI visitors were associated with reduced non-household within- and cross-ZIP code transmission in the same week (Fig. 4). Estimates of coefficients are provided in Extended Data Table 2. The model identifies a strong effect of vaccination on SARS-CoV-2 transmission: a 12.48% newly vaccinated population was associated with reductions of 28.0% (95% CI: 14.0% – 40.0%) and 14.8% (1.7% – 26.4%) for within- and cross-ZIP code non-household transmission events, respectively. In contrast, a 0.12 per capita increase of POI visitors was associated with increases of 9.6% (0.3% – 19.3%) and 14.4% (8.7% – 20.2%) for within- and cross-ZIP code transmission outside households, respectively. We further found that both within- and cross-ZIP code transmission had strong positive associations with log weekly cases per capita (. Higher percentage of Hispanic residents and lower cumulative cases per capita were associated with higher non-household transmission ( ). For cross-ZIP code transmission, cumulative cases per capita had a stronger effect than vaccination and POI visitors (Fig. 4b, Extended Data Table 2), indicating that prior infections may result in reduced cross-ZIP code transmission in locations with a higher attack rate. These findings reveal how health inequities related to COVID-19 manifest across NYC communities. Results also indicate that promoting vaccination and capacity limits or temporary limits on local businesses, schools, and other POIs in high-prevalence communities were effective in reducing SARS-CoV-2 transmission in NYC. These findings were corroborated with an alternate random-effect model (Supplementary Information), and testing of effect lags of one week and two weeks (Extended Data Figs. 6–8).

## Discussion

Here, leveraging detailed test and tracing data, we performed an analysis of ZIP code level SARS-CoV-2 transmission in NYC. The observed heterogeneity of SARS-CoV-2 spread at community scales implies that NPIs focusing on neighborhoods with extensive community transmission could potentially be more cost-effective. However, because communities with high test positivity were typically high poverty areas^3^, during isolation and quarantine resources (such as food delivery, medication delivery, and access to safe isolation places) should be provided to address the disproportionate impact of the pandemic on these communities. Our statistical analyses suggest that the combination of vaccination and reactive, zone-based intervention measures implemented in NYC likely reduced the spread of COVID-19 during the second wave.

This study has several limitations. Firstly, the contact tracing data were biased to household exposure, and voluntarily reported close contacts, especially outside the household, were incomplete. As a result, identified clusters of exposure and transmission are largely confined to small networks, limiting the detection of complete transmission networks, including super-spreading events. However, the spatial transmission pattern is less affected by the selection bias if such bias is similar across ZIP code areas. Secondly, some communities may have a lower response rate to the calls from tracers. Further studies are needed to quantify the factors associated with the lower response rate for improving future contact tracing effectiveness. Thirdly, due to missing and incorrect personal identifying information, the matching to close contacts and their test results may be incomplete.

With the global circulation of new variants of concern, such as Omicron and its sublinages^32^, our findings can inform control management in other urban settings beyond NYC. Specifically, public health authorities should consider the community-level spatial dispersion of SARS-CoV-2 when designing control tactics, which can be analyzed in real time using contact tracing data. Our analysis on the exposure network may inform a better definition of the proper geographical units for observation and interventions based on actual human interactions and disease transmission in NYC and elsewhere. Coordinated interventions targeting identified clusters of ZIP codes currently supporting the spatial transmission of SARS-CoV-2 could potentially produce more effective outbreak control. The findings may also support future pandemic preparedness and response. The operational performance of contact tracing can be used as a benchmark in urban settings and support modeling studies^33–36^ of the potential effects of contact tracing on emerging infectious disease containment.

## Supplementary Material

Supplement 1

## Data Availability

COVID-19 surveillance data in NYC at the MOZCTA (modified ZIP code tabulation area) level are publicly available at the GitHub repository maintained by the NYC Department of Health and Mental Hygiene (DOHMH) (https://github.com/nychealth/coronavirus-data). Demographic and socioeconomic data for NYC zip code tabulation areas (ZCTA) are available from the 5-year American Community Survey (ACS) (https://www.census.gov/programs-surveys/acs/data.html). Contact tracing records and individual testing results are subject to restrictions for the protection of patient privacy. Requests for data access should be addressed to NYC DOHMH and NYC Health + Hospitals.
