# Supplementary material for: Contact tracing reveals community transmission of COVID-19 in New York City
Source: Res Sq. 2022 Jul 27:rs.3.rs-1840065. Preprint. [Version 1] doi: 10.21203/rs.3.rs-1840065/v1 (PMC9347284; doi:10.21203/rs.3.rs-1840065/v1)
Supplement: Supplement 1 [file SIV2.docx]

**Supplementary Information**

Contact tracing reveals community transmission of COVID-19 in New York City

Sen Pei^1^*, Sasikiran Kandula^1^, Jaime Cascante-Vega^1^, Wan Yang^2^, Steffen Foerster^3^, Corinne Thompson^3^, Jennifer Baumgartner^3^, Shama Desai Ahuja^2,3^, Kathleen Blaney^3^, Jay K. Varma^4^, Theodore Long^5^, Jeffrey Shaman^1^

^1^Department of Environmental Health Sciences, Mailman School of Public Health, Columbia University, New York, NY, 10032, USA

^2^Department of Epidemiology, Mailman School of Public Health, Columbia University, New York, NY, 10032, USA

^3^New York City Department of Health and Mental Hygiene (DOHMH), Long Island City, NY, 11001, USA

^4^Department of Population Health Sciences, Weill Cornell Medical College, New York, NY, 10065, USA

^5^NYC Health + Hospitals, New York, NY

^*^Correspondence to: S.P. ([sp3449@cumc.columbia.edu](mailto:sp3449@cumc.columbia.edu))

# 1. Data

The NYC Test & Trace Corps (T2) initiative attempts to interview all confirmed and probable COVID-19 patients about their location and activities before and during the infectious period. Information about close contacts during the infectious period is elicited during the interview. Close contacts are then interviewed and monitored for the duration of their quarantine. The program offers resources to support quarantine and isolation free of charge, including food delivery, medication delivery, and access to hotels. Close contacts are encouraged to get tested.

We used T2 data collected in NYC from October 1, 2020 to May 10, 2021. The study period spans the second pandemic wave of COVID-19 in NYC. The data contain 5,735,726 phone call records of interactions between contact tracers and confirmed/probable cases and their contacts, as well as information gathered during the phone calls. Age and zip code of home location are available for most cases and contacts. Index cases and their contacts were identified in the dataset using a matching algorithm based on personal identifying information. We further cross-linked the contact tracing dataset with laboratory test results of COVID-19 in NYC during the same period to obtain the infection status of exposed persons. The daily numbers of contacted infected individuals in five age groups (0-9, 10-19, 20-49, 50-64, 65+) are shown in Extended Data Fig. 1a. During the study period, the age structure of confirmed cases remained stable; age group 20-49 accounted for most infections (Extended Data Fig. 1b). Use of this dataset in this study was approved by Columbia University Institutional Review Board (IRB) AAAT2182.

Demographic and socioeconomic data for NYC zip code tabulation areas (ZCTA) were compiled from the 5-year American Community Survey (ACS) (<https://www.census.gov/programs-surveys/acs/data.html>). Variables include population size, population density (persons per square kilometer), percentage of Black residents, percentage of Hispanic residents, percentage of population over 65 years old, median household income, percentage of residents with bachelor’s degree, and mean household size. We downloaded the 2019 estimates for these variables using the R package tidycensus^1^.

COVID-19 surveillance data in NYC at the MOZCTA (modified ZIP code tabulation area) level are available at the GitHub repository maintained by the NYC Department of Health and Mental Hygiene (DOHMH) (<https://github.com/nychealth/coronavirus-data>). We used weekly cases per capita, weekly tests per capita, and percentage of tests positive. Vaccination data were obtained from the public repository of DOHMH (<https://github.com/nychealth/covid-vaccine-data>). Human mobility data recording the weekly number of visitors to points of interest (POIs) in NYC were provided by SafeGraph (<https://safegraph.com/>), which aggregates anonymized location data from numerous mobile phone applications to provide insights about physical places, via the SafeGraph Community. To enhance privacy, SafeGraph excludes census block group information if fewer than five devices visited an establishment in a month from a given census block group. We aggregated the mobility data to zip code level to estimate the weekly number of visitors (regardless of visitors’ location of residence) to POIs in each zip code area. In the statistical analysis, we mapped the ACS data from the ZCTA level to the MOZCTA level to align the scale of the data. The mapping between ZCTA and MOZCTA is available at <https://data.cityofnewyork.us/Health/Modified-Zip-Code-Tabulation-Areas-MODZCTA-/pri4-ifjk>.

**2. Reconstructing transmission networks**

Due to asymptomatic and pre-symptomatic shedding, the reporting dates of index cases and contacts cannot be used to determine the direction of transmission. To address this issue, we developed a maximum-likelihood method to reconstruct transmission chains based on the risk of COVID-19 spread across different age groups. This approach includes three steps:

- 1. Estimate the infection time using symptom onset date or specimen collection date. Use the estimated infection time to determine the direction of exposure and transmission.
  2. Estimate the probability of transmission for exposures across age groups using test and trace data.
  3. Sample an ensemble of possible transmission networks and select the one that maximizes the transmission likelihood.

**2.1 Estimation of infection time**

For each pair of index case and contact, we inferred the direction of exposure or transmission using estimated infection time. All index cases were confirmed infections, but only a proportion of contacts were tested. We therefore used exposure pairs for which both the index case and contact had been tested and excluded exposure pairs for which contacts had not been tested, as these contacts did not affect the observed transmission network. If the contact tested negative, the direction of exposure is from the index case to the close contact (i.e., the index case is the infector); however, if the contact tested positive, the direction of exposure is uncertain and must be estimated.

For symptomatic cases who reported symptom onset dates, the infection time was estimated using the distribution of the incubation period reported from previous studies. Incubation period is the time between infection and symptom onset. Here we used a Weibull distribution estimated based on detailed contact tracing data from Hunan province, China^2^. Specifically, the probability density function (PDF) for the incubation distribution is

$$p\left( x \right)=\frac{k}{\lambda}\left( \frac{x}{\lambda} \right)^{k-1}e^{-\left( \frac{x}{\lambda} \right)^{k}}, [1]$$

where the shape parameter $k=1.58$ and the scale parameter $\lambda=7.11$. To estimate infection time, we randomly drew incubation periods (in days) for symptomatic cases from this PDF [1]. We also tested a log-normal incubation distribution estimated using contact tracing data from Shenzhen, China^3^. The transmission network remained similar.

For cases without symptoms, we used specimen collection date to estimate infection date. Denote $t_{inf\to test}$ as the interval from infection to specimen collection date. We aim to estimate $t_{inf\to test}$ given a person tested positive, i.e., $P\left( t_{inf\to test} | positive \right)$. Using Bayes’ rule, we have the following relation:

$$P\left( t_{inf\to test} | positive \right)\propto P\left( t_{inf\to test} \right)P\left( positive | t_{inf\to test} \right). [2]$$

Here $P\left( t_{inf\to test} \right)$ is the prior and $P\left( positive | t_{inf\to test} \right)$ is the likelihood of testing positive given that specimens were collected $t_{inf\to test}$ days after infection.

The prior $P\left( t_{inf\to test} \right)$ can be approximated using the interval from infection to specimen collection date for symptomatic cases, which provides a roughly plausible range of $t_{inf\to test}$. For each tested symptomatic case, $t_{inf\to test}$ is the sum of the sampled incubation period (from infection to symptom onset) and the time from symptom onset to specimen collection date, available in the dataset. The distribution of the prior $P\left( t_{inf\to test} \right)$ is shown in Extended Data Fig. 2a.

The likelihood $P\left( positive | t_{inf\to test} \right)$ was estimated using viral dynamics and limits of detection (LOD) for PCR tests. Following Larremore et al.^4^, we generated synthetic viral dynamics in infected persons. The log-transformed viral load (copies/mL), $V$, is a piece-wise linear function of the number of days after infection, $t$. Specifically, the viral dynamics is determined by three control points: $(t_{0}, 3)$, $(t_{peak}, V_{peak})$, and $(t_{f}, 6)$. Here $t_{0}$ is the time when log viral load reaches 3; $t_{peak}$ is the peak timing of log viral load; $V_{peak}$ is the peak magnitude of log viral load; and $t_{f}$ is the time when log viral load falls to 6. The mean log viral load was computed using the following function:

$$\bar{V}(t)=\left\{ \begin{aligned} \frac{3t}{t_{0}}, t\leq t_{0} \\ 3+\frac{\left( V_{peak}-3 \right)\left( t-t_{0} \right)}{t_{peak}-t_{0}}, t_{0}<t\leq t_{peak} \\ \max\left( V_{peak}-\frac{\left( V_{peak}-6 \right)\left( t-t_{peak} \right)}{t_{f}-t_{peak}},0 \right), t>t_{peak} \end{aligned} \right. [3]$$

In simulations, the following parameter distributions were used: $t_{0}\sim U(2.5, 3.5)$, $V_{peak}\sim U[7,11]$, $t_{peak}\sim\min\left( t_{0}+0.5+\Gamma\left( 1.5,1 \right), 3 \right)$, and $t_{f}\sim t_{peak}+U(2,6)$. Here $U(a,b)$ is a uniform distribution between $a$ and $b$; and$\Gamma(a,b)$ is a Gamma distribution with a shape parameter $a$ and a scale parameter $b$. The simulated log viral load on each day $t$ was drawn from a Gaussian distribution:

$$V\left( t \right)\sim N\left( \mu=\bar{V}\left( t \right), \sigma^{2}=0.04\bar{V}\left( t \right)^{2} \right). [4]$$

Sample trajectories of viral load are shown in Extended Data Fig. 2b.

We simulated 10^5^ viral load trajectories. For each trajectory, we randomly drew a LOD from $U[2,3.5]$, defined as the threshold for positive results – the test result is positive if $V(t)$ is above the LOD and negative otherwise. Using simulated viral dynamics and LOD, we obtained the likelihood $P\left( positive | t_{inf\to test} \right)$ as shown in Extended Data Fig. 2c.

We computed the posterior distribution $P\left( t_{inf\to test} | positive \right)$ using Eq. [2] (Extended Data Fig. 2d). The infection time for cases without symptoms was estimated using the distribution $P\left( t_{inf\to test} | positive \right)$. Once the infection time of index case - contact pairs had been sampled, using either symptom onset date or specimen collection date, the direction of exposure could be determined by the chronological order of infection.

**2.2 Estimation of transmission probability across age groups**

We further use the test and tracing date to estimate the transmission probability across age groups, which are used to reconstruct transmission chains. We classify the total population into four age groups: 0-9, 10-19, 20-64, and 65+. Denote $P_{a\to a^{'}}(positive)$ as the probability of successful transmission for an exposure from age group $a$ to $a'$. In actuality, we only observe $P_{a\to a^{'}}(positive|test)$ among tested exposures. Bayes’ rule gives

$$P_{a\to a^{'}}\left( positive \right)=P_{a\to a^{'}}\left( positive | test \right)\times\frac{P_{a\to a^{'}}\left( test \right)}{P_{a\to a^{'}}\left( test | positive \right)}, [5]$$

where $P_{a\to a^{'}}\left( test \right)$ is the probability that an exposure from age group $a$ to $a'$ is tested and $P_{a\to a^{'}}\left( test | positive \right)$ is the probability of testing given a successful transmission from age group $a$ to $a'$, i.e. an infection. A diagram for Eq. [5] is provided in Extended Data Fig. 3.

If we assume the relative test-seeking probability between exposed and infected individuals is independent of age, then $P_{a\to a^{'}}\left( test \right)/P_{a\to a^{'}}\left( test | positive \right)$ is constant across age groups. We then can use the test positivity rate for exposure from age group $a$ to $a'$, $P_{a\to a^{'}}\left( positive | test \right)$, to represent the relative transmission probability across age groups: $P_{a\to a^{'}}\left( positive \right)=\gamma P_{a\to a^{'}}\left( positive | test \right)$, where $\gamma=P_{a\to a^{'}}\left( test \right)/P_{a\to a^{'}}\left( test | positive \right)$. See Fig. S3 for more details.

To compute $P_{a\to a^{'}}(positive|test)$, we first used the method introduced in subsection 2.1 to determine the possible directions of exposure pairs for which both index case and contact were tested. Then we selected the pairs of exposures from age group $a$ to $a'$ and computed the probability of successful transmission for $a\to a^{'}$ exposures. We repeated this analysis 1,000 times and took the average transmission probability $P_{a\to a^{'}}(positive|test)$. Results are shown in Extended Data Table 1.

**2.3 Reconstruction of the maximum likelihood transmission network**

We combined the methods described in subsections 2.1 and 2.2 to reconstruct transmission networks. Using the method developed in subsection 2.1, we first estimated the possible directions of transmission events (in which both index case and contact tested positive) and used these directed transmission links to form a putative transmission network. For each transmission link $\mathcal{l}$, we identified the age groups for both patients (e.g., an exposure from age group $a$ to $a'$) and recorded the transmission probability across age groups for this link $\mathcal{l}$ in Extended Data Table 1 (estimated in subsection 2. 2) as $P_{\mathcal{l}}\left( positive | test \right)$. We computed the likelihood considering all transmission links: $L=\sqrt[n]{\prod_{\mathcal{l}} P_{\mathcal{l}}(positive|test)}$ where $\mathcal{l}$ runs over all $n$ transmission links in the network. We sampled 1,000 putative transmission networks and selected the network that maximizes the likelihood $L$ among the ensemble of possible transmission networks.

**3. Statistical analysis**

We used conditional autoregressive (CAR) models to analyze non-household within- and cross-ZIP code transmission in two separate models. The CAR model was implemented in a Bayesian hierarchical framework. Specifically, we fitted a Poisson generalized linear mixed model (GLMM) where the random effect was modeled by CAR priors to account for the inherent spatial-temporal autocorrelation present in the disease transmission data.

**3.1 Statistical model**

We modeled the numbers of non-household within- and cross-ZIP code transmission events using a modified Poisson generalized linear mixed model. Denote $y_{within}(i,t)$ and $y_{cross}(i,t)$ as the weekly numbers of non-household within-ZIP code and cross-ZIP code transmission events in ZIP code $i$ and week $t$. The week for transmission is determined by the self-reported contact time between index cases and contacts. Fixed effects include log-transformed population density, log-transformed weekly cases per capita, log-transformed weekly tests per capita, cumulative cases per capita, percentage of Black residents, percentage of Hispanic residents, percentage of population over 65 years old, median household income, percentage of residents with a bachelor’s degree, mean household size, percentage of fully vaccinated residents, and number of POI visitors per capita. All covariates were standardized to have mean zero and standard deviation one. We used log-transformed population as an offset.

Specifically, the model for non-household within-ZIP code transmission is described by the following equation:

$$\log\left( y_{within}(i,t+d) \right)=\log\left( population(i) \right)+\beta_{1}\times\log\left( population density(i) \right)+\beta_{2}\times\log\left( weekly cases per capita\left( i,t \right) \right)+\beta_{3}\times\log\left( weekly tests per capita\left( i,t \right) \right)+\beta_{4}\times cumulative cases per capita\left( i,t \right)+\beta_{5}\times\% Black resident\left( i \right)+\beta_{6}\times\% Hispanic resident\left( i \right)+\beta_{7}\times\% resident over 65\left( i \right)+\beta_{8}\times median household income\left( i \right)+\beta_{9}\times\% bachelor^{'}s degree\left( i \right)+\beta_{10}\times mean household size\left( i \right)+\beta_{11}\times\% fully vaccinated resident\left( i, t \right)+\beta_{12}\times weekly POI visitors per capita\left( i,t \right)+\psi_{it}+\varepsilon_{it}. [6]$$

Here $d$ is the lag (in weeks), $\log\left( population(i) \right)$ is the offset, $\psi_{it}$ is the random effect for location $i$ and week $t$, and $\varepsilon_{it}$ is the error term. In the main model, we used $d=0$ (no lag). We additionally tested $d=1$ and $d=2$ as a sensitivity analysis.

The model for cross-zip code transmission is defined similarly:

$$\log\left( y_{cross}(i,t+d) \right)=\log\left( population(i) \right)+\beta_{1}\times\log\left( population density(i) \right)+\beta_{2}\times\log\left( weekly cases per capita\left( i,t \right) \right)+\beta_{3}\times\log\left( weekly tests per capita\left( i,t \right) \right)+\beta_{4}\times cumulative cases per capita\left( i,t \right)+\beta_{5}\times\% Black resident\left( i \right)+\beta_{6}\times\% Hispanic resident\left( i \right)+\beta_{7}\times\% resident over 65\left( i \right)+\beta_{8}\times median household income\left( i \right)+\beta_{9}\times\% bachelor^{'}s degree\left( i \right)+\beta_{10}\times mean household size\left( i \right)+\beta_{11}\times\% fully vaccinated resident\left( i, t \right)+\beta_{12}\times weekly POI visitors per capita\left( i,t \right)+\psi_{it}+\varepsilon_{it}. [7]$$

**3.2 Controlling for spatial-temporal autocorrelation**

We first fitted a model without considering spatial-temporal autocorrelation in the random effect $\psi_{it}$; however, we found signatures of spatial autocorrelation in the residuals using Moran’s I test for each week. The dependent variables are also temporally autocorrelated. Neglecting such spatial-temporal autocorrelation will lead to overconfidence in the estimated effect size, i.e., the standard errors will be biased too small^5^.

To account for this inherent spatial-temporal autocorrelation in the dependent variables $y_{within}(i,t)$ and $y_{cross}(i,t)$, we modeled the random effect $\psi_{it}$ using conditional autoregressive (CAR) priors^6,7^. Specifically, we used the CAR model proposed by Rushworth et al.^8^, which represents the spatial-temporal structure as a multivariate autoregressive process with a spatially autocorrelated precision matrix. The model is specified by^6^:

$$\psi_{it}=\phi_{it}, [8]$$

$$\boldsymbol{\phi}_{t}|\boldsymbol{\phi}_{t-1}\sim N\left( \rho_{T}\phi_{t-1},\tau^{2}\boldsymbol{Q}\left( \boldsymbol{W},\rho_{S} \right)^{-1} \right), t=2, \ldots, T$$

$$\boldsymbol{\phi}_{1}\sim N\left( \boldsymbol{0}, \tau^{2}\boldsymbol{Q}\left( \boldsymbol{W},\rho_{S} \right)^{-1} \right),$$

$$\tau^{2}\sim Inverse-Gamma\left( a,b \right),$$

$$\rho_{S},\rho_{T}\sim Uniform\left( 0,1 \right).$$

In this model $\boldsymbol{\phi}_{t}=(\phi_{1t}, \ldots, \phi_{Kt})$ is the vector of random effects for time $t$, which evolve over time via a multivariate first order autoregressive process with temporal autoregressive parameter $\rho_{T}$. The spatial autocorrelation is induced by the variance $\tau^{2}\boldsymbol{Q}\left( \boldsymbol{W},\rho_{S} \right)^{-1}$. The precision matrix $\boldsymbol{Q}\left( \boldsymbol{W},\rho_{S} \right)$ depends on the spatial adjacency matrix $\boldsymbol{W}$ and the spatial autoregressive parameter $\rho_{S}$. We used a binary spatial adjacency matrix $\boldsymbol{W}=(w_{ij})$, where the entry $w_{ij}$ is one if location $i$ and location $j$ share a common border and is zero otherwise. Additionally, $w_{ii}=0$. The functional form of $\boldsymbol{Q}\left( \boldsymbol{W},\rho_{S} \right)$ is given by^9^

$$\boldsymbol{Q}\left( \boldsymbol{W},\rho_{S} \right)=\rho_{S}\left[ diag\left( \boldsymbol{W}\boldsymbol{1} \right)-\boldsymbol{W} \right]+\left( 1-\rho_{S} \right)\boldsymbol{I,}$$

where $\boldsymbol{1}$ is the $K\times1$ vector of ones and $\boldsymbol{I}$ is the $K\times K$ identity matrix.

We implemented the model using the function ST.CARar in the R package CARBayesST^10^. Using a Bayesian hierarchical framework, model coefficients in Eqs. [6-7] and parameters in Eq. [8] were estimated using a Markov chain Monte Carlo (MCMC) algorithm^11^. We fitted the model using data from 177 MOZCTAs and 31 weeks ($K=177, T=31)$. In the main model, we used $d=0$ (no lag). We generated 420,000 MCMC samples for each coefficient/parameter and discarded the first 20,000 samples as the burn-in period. The remaining samples were subsequently thinned by 20 to reduce the autocorrelation of the Markov chain. In total, 20,000 MCMC samples were generated for each coefficient and parameter. The convergence of Markov chains was diagnosed using the convergence diagnostic proposed by Geweke^12^. The diagnostic statistics for all coefficients and hyperparameters were within the range (-1.96, 1.96), suggesting convergence of Markov chains.

We further evaluated the spatial-temporal autocorrelation in the model residuals to confirm that spatial and temporal structures had been annihilated. Specifically, we examined the spatial autocorrelation in residuals from the 177 locations in each week using Moran’s I. The spatial autocorrelation of the residuals was absent for most weeks ($p>0.05$) (Extended Data Fig. 4). For each location, we evaluated the temporal autocorrelation in the residuals using the Durbin-Watson test. Results indicate that temporal autocorrelation was not significant for most locations ($p>0.05$) (Extended Data Fig. 5).

The effects of covariates are represented by the exponentiated coefficient, or the incidence rate ratio. The incidence rate ratio quantifies the multiplicative change in the number of transmission events if each covariate increases by one standard deviation, adjusting for all other covariates in the model. The distributions of incidence rate ratios were obtained from the 20,000 MCMC samples. The median, 95% CI, and p-values were derived from these empirical distributions.

A few sensitivity analyses were performed to assess the robustness of the results. First, we additionally tested one-week and two-week lags. The qualitative results remained similar (Extended Data Figs. 6-7). Second, we used another form of the random effect model proposed by Knorr-Held et al.^13^. In this model, the spatial-temporal variation in the data is decomposed into three components: an overall spatial effect common to all time periods, an overall temporal trend common to all spatial units, and a set of independent space-time interactions. See more details in Ref.^6^. The model was fitted using the function ST.CARanova in the R package CARBayesST. A zero-week effect lag was used and the MCMC setting was the same. Results hold as in the main model (Extended Data Fig. 8).

# Reference

1. Walker, K., Herman, M. & Eberwein, K. *tidycensus: Load US Census Boundary and Attribute Data as ‘tidyverse’ and ’sf’-Ready Data Frames*. (2021).

2. Hu, S. *et al.* Infectivity, susceptibility, and risk factors associated with SARS-CoV-2 transmission under intensive contact tracing in Hunan, China. *Nat. Commun.* **12**, 1533 (2021).

3. Bi, Q. *et al.* Epidemiology and transmission of COVID-19 in 391 cases and 1286 of their close contacts in Shenzhen, China: a retrospective cohort study. *Lancet Infect. Dis.* **20**, 911–919 (2020).

4. Larremore, D. B. *et al.* Test sensitivity is secondary to frequency and turnaround time for COVID-19 screening. *Sci. Adv.* eabd5393 (2020) doi:10.1126/sciadv.abd5393.

5. F. Dormann, C. *et al.* Methods to account for spatial autocorrelation in the analysis of species distributional data: a review. *Ecography* **30**, 609–628 (2007).

6. Lee, D., Rushworth, A. & Napier, G. Spatio-Temporal Areal Unit Modeling in R with Conditional Autoregressive Priors Using the CARBayesST Package. *J. Stat. Softw.* **84**, 1–39 (2018).

7. Lee, D. CARBayes: an R package for Bayesian spatial modeling with conditional autoregressive priors. *J. Stat. Softw.* **55**, 1–24 (2013).

8. Rushworth, A., Lee, D. & Mitchell, R. A spatio-temporal model for estimating the long-term effects of air pollution on respiratory hospital admissions in Greater London. *Spat. Spatio-Temporal Epidemiol.* **10**, 29–38 (2014).

9. Leroux, B. G., Lei, X. & Breslow, N. Estimation of Disease Rates in Small Areas: A new Mixed Model for Spatial Dependence. in *Statistical Models in Epidemiology, the Environment, and Clinical Trials* (eds. Halloran, M. E. & Berry, D.) 179–191 (Springer, 2000). doi:10.1007/978-1-4612-1284-3_4.

10. Lee, D., Rushworth, A. & Pettersson, G. N. and W. *CARBayesST: Spatio-Temporal Generalised Linear Mixed Models for Areal Unit Data*. (2021).

11. Gelman, A., Carlin, J. B., Stern, H. S. & Rubin, D. B. *Bayesian Data Analysis*. (Chapman and Hall/CRC, 1995). doi:10.1201/9780429258411.

12. Geweke, J. Evaluating the Accuracy of Sampling-Based Approaches to the Calculation of Posterior Moments. in *In Bayesian Statistics* 169–193 (University Press, 1992).

13. Knorr-Held, L. Bayesian modelling of inseparable space-time variation in disease risk. *Stat. Med.* **19**, 2555–2567 (2000).

**Extended Data**

Extended Data Fig. 1. Age structure of index cases called by contact tracers. (a) The daily number of index cases in each age group from October 1 2020 to May 10 2021. There was a data reporting issue during March 2021 so the spike in March does not reflect the actual COVID-19 situation. (b) The proportion of index cases in each age group during the study period.

Extended Data Fig. 2. Estimating the distribution of the interval from infection to testing for positive cases. (a) The prior distribution of $t_{inf\to test}$ obtained using data from symptomatic infections. (b) Samples of synthetic viral load trajectories. (d). The likelihood $P(positive|t_{inf\to test})$ obtained using synthetic viral dynamics and LOD. (d). The posterior $P(t_{inf\to test}|positive)$.


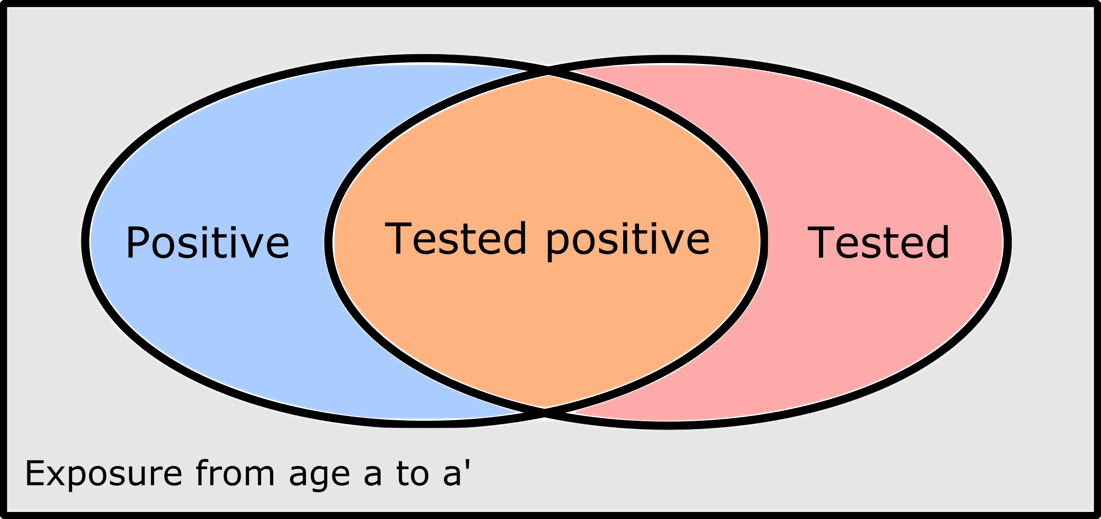


Extended Data Fig. 3. A diagram for estimating the transmission probability for an exposure from age group $a$ to $a'$. Assume the grey rectangular has an area of 1, representing all exposures from age group $a$ to $a'$. The combined orange and red areas represent the probability of testing, $P_{a\to a^{'}}(test)$; the combined orange and blue areas represent the probability of infection, $P_{a\to a^{'}}(positive)$; the ratio of the orange area to the combined orange and red areas is the positivity rate, $P_{a\to a^{'}}\left( positive | test \right)=orange/(orange+red)$; the ratio of the orange area to the combined orange and blue areas is the probability of testing among infected exposures, $P_{a\to a^{'}}\left( test | positive \right)=orange/(orange+blue)$. Since the orange area can be computed by $P_{a\to a^{'}}\left( test \right)\times P_{a\to a^{'}}\left( positive | test \right)$, or $P_{a\to a^{'}}\left( positive \right)\times P_{a\to a^{'}}\left( test | positive \right)$, we have $P_{a\to a^{'}}\left( test \right)\times P_{a\to a^{'}}\left( positive | test \right)=P_{a\to a^{'}}\left( positive \right)\times P_{a\to a^{'}}\left( test | positive \right)$. Then we can estimate the transmission probability for an exposure from age group $a$ to $a'$ through $P_{a\to a^{'}}\left( positive \right)=P_{a\to a^{'}}\left( test \right)\times P_{a\to a^{'}}\left( positive | test \right)/P_{a\to a^{'}}\left( test | positive \right)$.

Extended Data Fig. 4. Moran’s I test for residual spatial autocorrelation. Moran’s I test was performed for each week. P-values for within- and cross-zip code transmission are shown. The horizontal dash line marks the p=0.05 threshold.

Extended Data Fig. 5. Durbin-Watson test for residual temporal autocorrelation performed for each location. P-values for within- and cross-zip code transmission are shown. The horizontal dash line marks the p=0.05 threshold.

Extended Data Fig. 6. Results of sensitivity analysis using a one-week lag. Dots and horizontal lines show median values and 95% CIs. DIC=6,301 for a and DIC=12,555 for b.

Extended Data Fig. 7. Results of sensitivity analysis using a two-week lag. Dots and horizontal lines show median values and 95% CIs. DIC=6,257 for a and DIC=12,401 for b.

Extended Data Fig. 8. Results of sensitivity analysis using an alternate random effects model form. No lag effect was used. Dots and horizontal lines show median values and 95% CIs. DIC=6,349 for a and DIC=12,641 for b.

|  | **0-9** | **10-19** | **20-64** | **65+** |
| --- | --- | --- | --- | --- |
| **0-9** | 0.507 | 0.499 | 0.457 | 0.516 |
| **10-19** | 0.469 | 0.472 | 0.436 | 0.530 |
| **20-64** | 0.470 | 0.440 | 0.346 | 0.482 |
| **65+** | 0.457 | 0.449 | 0.388 | 0.604 |

Extended Data Table 1. Transmission probability $P_{a\to a^{'}}\left( positive | test \right)$ across age groups. Row indicates the age group of index cases, and column indicates the age group of contacts.

| **Variables** | **Non-household within-ZIP code** | **Non-household cross-ZIP code** |
| --- | --- | --- |
| POI visitors per capita | 0.0914 (0.0031, 0.1765) | 0.1346 (0.0839, 0.1842) |
| % fully vaccinated residents | -0.3281 (-0.5100, -0.1503) | -0.1600 (-0.3066, -0.0173) |
| Mean household size | 0.0700 (-0.0573, 0.1981) | -0.0798 (-0.1684, 0.0077) |
| % residents with bachelor | 0.1298 (-0.0660, 0.3394) | 0.0852 (-0.0411, 0.2138) |
| Median household income | -0.2342 (-0.4034, -0.0714) | -0.0564 (-0.1564, 0.0432) |
| % 65+ population | -0.0218 (-0.1132, 0.0703) | -0.0640 (-0.1227, -0.0066) |
| % Hispanic residents | 0.1286 (0.0405, 0.2149) | 0.1244 (0.0562, 0.1946) |
| % Black residents | -0.0375 (-0.1207, 0.0438) | 0.0947 (0.0398, 0.1527) |
| Cumulative case per capita | -0.1902 (-0.3448, -0.0364) | -0.2349 (-0.3671, -0.1116) |
| Log(weekly test per capita) | -0.0537 (-0.1506, 0.0426) | 0.0022 (-0.0609, 0.0639) |
| Log(weekly case per capita) | 0.9508 (0.8176, 1.0864) | 0.7761 (0.6817, 0.8666) |
| Log(population density) | -0.0782 (-0.1695, 0.0165) | 0.0107 (-0.0528, 0.0770) |

Extended Data Table 2. Estimated coefficients for the main model. Results show the median estimates and 95% CIs. Significant covariates (p<0.05) are highlighted in blue.
